# Recommendation on unbiased estimation of population attributable fraction calculated in “prevalence and risk factors of active pulmonary tuberculosis among elderly people in China: a population based cross-sectional study”

**DOI:** 10.1186/s40249-019-0587-8

**Published:** 2019-08-19

**Authors:** Ahmad Khosravi, Mohammad Ali Mansournia

**Affiliations:** 1 Department of Epidemiology, School of Public Health, Shahroud University of Medical Sciences, Shahroud, Iran; 2 Department of Epidemiology and Biostatistics, Tehran University of Medical Sciences, Tehran, Iran

**Keywords:** Population attributable fraction, Confounding, Sample-weighted parametric g-formula

## Abstract

Population attributable fraction (PAF) refers to the proportion of all cases with a particular outcome in a population that could be prevented by eliminating a specific exposure. The authors of a recent paper evaluated the prevalence and estimated the PAFs for risk factors of TB among elderly people in China [Inf Dis Poverty. 2019;8:7]. Confounding is inevitable in observational studies and Levin’s formula is of limited use in practice for unbiasedly estimating PAF. In a complex survey design, an unbiased estimation of the PAF can be calculated using a sample-weighted version of the Miettinen formula or a sample weighed parametric g-formula. With respect to causal interpretation of PAF in public health setting, computation of PAF is logical and practical when the exposure is amenable to intervention.

**Electronic supplementary material:**

The online version of this article (10.1186/s40249-019-0587-8) contains supplementary material, which is available to authorized users.

## Multilingual abstracts

Please see Additional file 1 for translations of the abstract into the five official working languages of the United Nations.

**To the Editor.**

We read with great interest a recent article titled [1]: “Prevalence and risk factors of active pulmonary tuberculosis among elderly people in China: a population based cross-sectional study”. The authors evaluated the prevalence and identify the risk factors of TB among elderly people in China using a cross-sectional study. However, there are several concerns in the analysis.

i). In the statistical analysis section it was indicated that population attributable fraction (PAF) of each adjusted risk factor was estimated using Levin’s formula where *RR* is the risk ratio and *p*_*e*_ means proportion of population exposed to risk factors [2].

1 $$ PAF=\frac{p_e\left( RR-1\right)}{p_e\left( RR-1\right)+1} $$

In the study, the adjusted odds ratio (*OR*) was used in place of *RR*.

PAF refers to the proportion of all cases with a particular outcome in a population that could be prevented by eliminating a specific exposure [3]. Formula 1 is unbiased in the absence of confounding and effect modification [3, 4]. Observational studies are subject to confounding which will lead to bias if Levin’s formula is inappropriately applied to estimate PAFs [3]. The Levin’s formula is valid only for unadjusted risk ratio [3–5]. The bias from this error will depend on the degree of confounding [6]. For a dichotomous exposure an unbiased estimation of PAF can be calculated using the Miettinen’s formula [7].

2 $$ PAF=\frac{p_c\left({RR}_{adj}-1\right)}{RR_{adj}} $$

Where *RR*_*adj*_ is the adjusted risk ratio and *p*_*c*_ is the prevalence of exposure among the cases. This produces valid estimate in the presence of confounding, assuming exposure status and confounders are accurately measured and adjusted for.

As an example, in this study [1] the adjusted *OR* and prevalence of diabetes in the active TB cases was reported 1.83 (1.08–3.10) and 16/193, respectively. The PAF using formula 2 is 3.76% which is less than the reported value (5.52%).

ii). The term “attributable” refers to a causal interpretation [3]. One of the main assumptions underlying the PAF is no bias in study design. Therefore, the application of formula 2 in cohort design is acceptable but for case-control and cross-sectional studies, it needs more considerations. In a cross-sectional study, reverse causality and prevalence-incidence bias are the main concerns for assessing the effect of the exposure on the outcome.
iii). Another potential source of bias in the study is failure to adjust observed estimates of the prevalence of TB and exposure to risk factors for the complex sampling design employed. With such a design, the population prevalence should be adjusted using inverse probability weighting (IPW) so that the reported prevalence is appropriately adjusted for multistage and disproportionate sampling [8]. Further, the authors do not mention whether or how clustering was taken account of in the multivariable logistic regression modeling.
iv). For complex survey designs, it is necessary to adjust PAFs for the complex sampling design [9, 10]. PAF can be computed as a sample-weighted version of the Miettinen or Bruzzi formula (formula 2 in reference [9] or formula 3 in reference [10] or sample-weighted model-based standardization, also known as parametric g-formula (formula 3 in reference [9] or formula 4 in reference [10].
v). As PAF is a function of the prevalence of exposure, self-reported measurement of exposures in this study can be lead to bias in the estimation of PAF. As reported in this study, the self-reported and local health documentation search of diabetes was not sufficient to estimate the real distribution.
vi). With respect to causal interpretation of PAF in a public health setting, computation of PAF is logical and practical when the exposure is amenable to intervention [6]. Therefore, it is less apparent why the attributable fraction for unmodifiable risk factors such as age and sex may be of use.

In sum, unbiased estimation of PAF requires several assumptions which are often ignored in practice. We recommend using sample-weighted version of Miettinen formula or sample weighed parametric g-formula [3, 11].

## Additional file

Additional file 1: Multilingual abstracts in the five official working languages of the United Nations. (PDF 485 kb)

## Data Availability

Not applicable.

## Abbreviation

PAF: Population attributable fraction
