# Supplementary material for: Recommendation on unbiased estimation of population attributable fraction calculated in “prevalence and risk factors of active pulmonary tuberculosis among elderly people in China: a population based cross-sectional study”
Source: Infect Dis Poverty. 2019 Aug 19;8:75. doi: 10.1186/s40249-019-0587-8 (PMC6699105; doi:10.1186/s40249-019-0587-8)
Supplement: Supplementary file 1 — Multilingual abstracts in the five official working languages of the United Nations. (PDF 485 kb) [file 40249_2019_587_MOESM1_ESM.pdf]

Translation of the abstract into the five official working languages of the United Nations

توصية بشأن التقدير غير المتحيز للجزء المنسوب للسكان المحسوب في "عوامل انتشار ومخاطر السل الرئوي النشط بين كبار السن في الصين: دراسة مقطعية قائمة على السكان"

أحمد خصرافي، محمد علي منصورنية

الملخص:
الجزء المنسوب للسكان (PAF) هو نسبة الحالات التي انتهى بها الأمر الى نتيجة معينة في فئة ما وكان بالامكان تلافي تلك النتيجة عبر اقصاء تعرض معين. قيم باحثو دراسة حديثة مدى التفشي وقدروا الأجزاء المنسوب للفئة PAFs لعوامل خطر الاصابة بالسل بين كبار السن في الصين 2019;8:7. [Inf Dis Poverty.2019;8:7]]. الارتباك حتمي في الدراسات القائمة على المراقبة واستخدام صيغة ليفين محدود من الناحية العملية في تقدير الجزء المنسوب للفئة PAF تقديرا غير منحاز . في تصميمات المسح المعقدة، يمكن تقدير الجزء المنسوب للفئة PAF تقديرا غير منحاز باستعمال نسخة من صيغة ميتينين Miettinen تقوم على العينات الموزونة أو صيغة g-formula باراميترية تقوم على العينات الموزونة. مع احترامنا لتفسير الجزء المنسوب للعامة تفسيرا سببيا في مجال الصحة العمومية، الا أن تقديره منطقي وعملي اذا ما كان التعرض قابل للاقصاء عن طريق التدخل.

Translated from English version into Arabic by Firas Arfaoui, proofread by Maysaa Abed, through

TRANSLATORS
WITHOUT BORDERS

对“Prevalence and risk factors of active pulmonary tuberculosis among elderly people in China: a population based cross-sectional study”一文关于人群归因分值无偏估计的建议

Ahmad Khosravi, Mohammad Ali Mansournia

摘要：
人群归因分值(population attributable fraction，PAF)是指在一个人群的特定时期内，如果消除或者预防某（些）确定的危险因素后（同时其它危险因素在人群中的暴露分布保持不变），所获得的人群中疾病发生风险降低的比例。近期 IDP 发表的一篇论文的作者评估了中国老年人中肺结核的患病率和人群归因危险分值 [Inf Dis Poverty. 2019;8:7]。在观察性研究中，混杂变量是不可避免的，而且 Levin 公式在实践中用于无偏估计 PAF 的用途有一定局限性。在复杂的调查设计中，可以使用 Miettinen 公式的样本加权版本或样本加权参数 g-公式来计算 PAF 的无偏估计。在公共卫生环境中应用 PAF 进行因果解释，当暴露适合干预时，PAF 的计算是合乎逻辑且具有实际意义。

Translated from English version into Chinese by Xin-Yu Feng, edited by Pin Yang

CDC CHINESE CENTER FOR DISEASE CONTROL AND PREVENTION
NATIONAL INSTITUTE OF PARASITIC DISEASES

Recommandation sur l'estimation sans biais de la fraction attribuable dans la population, calculée dans «Prevalence and risk factors of active pulmonary tuberculosis among elderly people in China:

a population based cross-sectional study » (prévalence et facteurs de risque de la tuberculose pulmonaire active chez les personnes âgées en Chine : une étude transversale basée sur la population)

Ahmad Khosravi, Mohammad Ali Mansournia

Résumé

La fraction attribuable dans la population (FAP) désigne la proportion de tous les cas présentant un résultat particulier dans une population et qui pourraient être prévenus par l'élimination d'une exposition spécifique. Les auteurs d'un article récent ont évalué la prévalence et estimé les FAP pour les facteurs de risque de tuberculose chez les personnes âgées en Chine [Inf Dis Poverty. 2019;8:7]. La confusion est inévitable dans les études observationnelles et la formule de Levin est d'une utilité limitée dans la pratique pour estimer sans biais la FAP. Dans un plan d'enquête complexe, une estimation sans biais de la FAP peut être calculée à l'aide d'une version à échantillon pondéré de la formule de Miettinen ou d'une formule-g paramétrique à échantillon pondéré. En ce qui concerne l'interprétation causale de la FAP dans le milieu de la santé publique, le calcul de la FAP est logique et pratique lorsque l'exposition peut faire l'objet d'une intervention.

Translated from English version into French by Allegra Guez, proofread by Maëva Paoletti through

TRANSLATORS
WITHOUT BORDERS

Рекомендации по объективной оценке дополнительной доли в популяции, рассчитанной в «Распространенность и факторы риска активного туберкулеза легких среди пожилых людей в Китае: поперечное исследование среди населения»

Ахмад Хосрави, Мохаммад Али Мансурия

Аннотация:

Дополнительная доля в популяции (ДДП) относится к доле всех случаев с определенным результатом в популяции, которые могут быть предотвращены путем устранения конкретного воздействия. Авторы недавно опубликованной работы оценили распространенность и подсчитали ДДП для факторов риска туберкулеза легких среди пожилых людей в Китае [Inf Dis Poverty. 2019;8:7]. В обсервационных исследованиях неизбежно смешивание, а формула Левина имеет ограниченное практическое применение для объективной оценки ДДП. В сложной схеме обследования объективная оценка дополнительной доли в популяции может быть рассчитана по формуле Миеттинена в варианте с использованием взвешенной выборки или по параметрической g-формуле для взвешенной выборки. Что касается причинной интерпретации ДДП в системе общественного здравоохранения, то расчеты ДДП являются логичными и практичными, когда воздействие подвергается вмешательству.

Translated from English version into Russian by Aleksandra Sadovnikova, proofread by Alexander Somin, through

Recomendación sobre la estimación imparcial de la fracción atribuible de población calculada en "Prevalencia y factores de riesgo de tuberculosis pulmonar activa en personas de edad avanzada en China: un estudio transversal basado en la población"

Ahmad Khosravi, Mohammad Ali Mansournia

Resumen:

La fracción atribuible a la población (FAP) se refiere a la proporción de todos los casos con un resultado particular, en una población que podría prevenirse, eliminando una exposición específica. Los autores de un artículo reciente evaluaron la prevalencia y estimaron las FAP,por factores de riesgo de tuberculosis, entre las personas mayores en China [Inf Dis Poverty. 2019;8:7]. La confusión es inevitable, en los estudios observacionales y la fórmula de Levin tiene un uso limitado, en la práctica, para estimar de manera imparcial FAP. En un diseño de encuesta complejo, se puede calcular una estimación no sesgada de FAP, utilizando una versión ponderada de la muestra de la fórmula de Miettinen o una fórmula-g paramétrica ponderada de la muestra. Con respecto a la interpretación causal de FAP, en el entorno de salud pública, el cálculo de FAP es lógico y práctico, si la exposición es susceptible de intervención.

Translated from English version into Spanish by María Luz Puerta, proofread by Rosanna Lenci, through
